# Exploring the Clinical Characteristics and Outcomes of Rhinovirus Infection in Hospitalized Children Compared with Other Respiratory Viruses

**DOI:** 10.3390/children11111303

**Published:** 2024-10-28

**Authors:** Sigrid Covaci, Claudiu Filimon, Mihai Craiu

**Affiliations:** 1 Carol Davila University of Medicine and Pharmacy, 050474 Bucharest, Romania; sigrid_covaci@yahoo.ca (S.C.); mcraiu@yahoo.com (M.C.); 2 National Institute for Mother and Child Health, Alessandrescu-Rusescu, 020395 Bucharest, Romania

**Keywords:** rhinovirus, children, hospitalized, influenza, RSV, SARS-CoV-2, respiratory viruses

## Abstract

Background: Acute viral respiratory tract infections constitute a significant challenge in pediatric healthcare globally, with rhinovirus representing one of the primary etiological agents. In this context, we conducted a study with the objective of identifying the clinical characteristics and outcomes of rhinovirus infection in comparison with other respiratory viruses in children hospitalized in one of the largest pediatric hospitals in the capital of Romania. Methods: We conducted a retrospective study among children hospitalized for influenza-like illness symptoms and who were tested by multiplex RT-PCR with a nasopharyngeal swab between May 2020 and December 2021. Results: A total of 496 children were eligible for inclusion in the study, and the positivity rate for at least one virus was 58.5%. The rhinovirus was identified in 138 patients (median age 12.5 months), representing 27.8% of all children tested and 49.3% of all positive samples. Although the clinical features of children with rhinovirus were dominated by cough (63.7%) and dyspnea (51.6%), no symptoms were identified that were strongly associated with rhinovirus infection in comparison to other respiratory viruses. The probability of receiving an antibiotic prescription was 1.92 times lower (*p* = 0.011) in children who tested positive for rhinovirus compared to children with negative RT-PCR results. The incidence of acute bronchiolitis or acute bronchitis, acute respiratory failure, and acute otitis media was higher among rhinovirus-positive children than among those who tested negative via RT-PCR. However, the incidence of these conditions was similar among children who tested positive for other respiratory viruses. Conclusions: Rhinovirus was the most prevalent virus identified in children hospitalized with influenza-like illness symptoms. The utilization of multiplex RT-PCR molecular tests is instrumental in elucidating etiology with precision and implementation of these advanced diagnostic methods, which can bring significant benefits in practice. A positive result for rhinovirus helps to reduce the unnecessary administration of antibiotics and optimizes patient management, thus decreasing the risk of severe complications such as acute respiratory failure and acute otitis media.

## 1. Introduction

Acute respiratory tract infections caused by viruses represent a substantial challenge to global health systems, ranking among the most common reasons for seeking medical care among both adults and children [1,2]. In the past, rhinovirus infections were classified as mild and self-limiting. However, advances in molecular diagnostic techniques have revolutionized our understanding of these infections. It has been found that rhinoviruses not only affect the upper respiratory tract but can also cause severe lower respiratory tract complications such as acute bronchitis, bronchiolitis, or pneumonia [3]. In light of these considerations, a reassessment of the role of rhinoviruses in respiratory diseases is imperative for the advancement of more efficacious prevention and management strategies. This is particularly crucial given the potential of these viruses to contribute to increased morbidity, particularly among vulnerable groups such as children, the elderly, and individuals with pre-existing comorbidities [4,5].

Rhinovirus, a member of the *Picornaviridae* family, is distinguished by its exceptionally small size, approximately 30 nm, which enables rapid and efficient transmission. The virus has a single-stranded RNA genome and exhibits numerous similarities with other enteroviruses [6,7]. It is highly contagious, transmitted by aerosols and direct contact, and exhibits resistance to dry environments and low temperatures. This latter characteristic facilitates rapid and straightforward replication within the upper respiratory tract [8]. Currently, three distinct rhinovirus species, designated as A, B, and C, have been identified, collectively comprising approximately 160 distinct serotypes. These serotypes are distinguished from one another on the basis of variations in surface glycoprotein and diversity in viral RNA structures [9]. A significant feature of rhinovirus biology is the absence of error-correcting mechanisms in RNA genome replication, which results in a high frequency of mutations. This high mutation rate contributes to the extensive antigenic variability of the virus, which presents a significant challenge in the development of an effective vaccine [9]. Due to the genetic diversity of rhinoviruses, the host immune response is evaded, resulting in recurrent infections and posing a significant challenge to the control strategies employed for viral respiratory infections [5,10]. Rhinovirus exhibits a marked affinity for the epithelial cells of the respiratory tract, initiating infection in the nasal or conjunctival mucosa and subsequently spreading to the posterior nasopharynx and tracheobronchial epithelium [6]. The interaction between rhinovirus and the host is influenced by a number of factors, including the individual’s immune status, age, the presence of comorbidities, and the genetic variability of the virus. These variables can result in a wide range of clinical manifestations, from self-limiting or even asymptomatic infections to severe complications [11].

In this context, we conducted a comprehensive study aimed at identifying and analyzing the distinctive clinical characteristics and outcomes of rhinovirus infection in comparison with other respiratory viruses among children hospitalized in one of the largest pediatric hospitals in the Romanian capital. This study brings new insights by focusing on the specific clinical patterns and complications associated with rhinovirus and by comparing its impact with that of other respiratory pathogens, providing a clearer understanding of its role in pediatric morbidity. 

## 2. Materials and Methods

A retrospective study was conducted among children hospitalized for influenza-like illness (ILI) symptoms and who were tested by multiplex RT-PCR via nasopharyngeal swabs between May 2020 and December 2021. All cases included in the analysis were admitted to the National Institute for Mother and Child Health (NIMCH) “Alessandrescu-Rusescu” in Bucharest, a tertiary care facility with a specialized focus on pediatrics and obstetrics–gynecology. The NIMCH is one of Romania’s major medical institutions, with over 70,000 pediatric patients hospitalized or undergoing emergency evaluation annually [12]. During the period under review, the NIMCH concentrated its efforts on the care of non-COVID-19 patients, with a particular focus on those with respiratory conditions. This was made possible by the availability of a multiplex RT-PCR testing machine, which was introduced in May 2021.

The study population comprised all patients younger than 18 years of age who were hospitalized for at least 24 h due to an acute illness with onset within the last seven days and who exhibited at the time of admission at least one symptom from the ILI definition [13], and who were tested by multiplex RT-PCR via a nasopharyngeal swab on the first day of hospitalization. Patients with a symptom onset of more than seven days, a positive RT-PCR result for at least one of the bacteria, or in whom other laboratory investigations proved bacterial etiology were excluded. In addition, patients with incomplete or uncertain data in their medical records and those tested more than 24 h after hospitalization were excluded.

All patients included in the study underwent testing via nasopharyngeal swab using the same QIAstat-Dx^®^ Respiratory SARS-CoV-2 Panel multiplex RT-PCR kit (Qiagen, Hilden, Germany). Consequently, a total of 21 respiratory pathogens were tested (viruses: influenza A with subtypes H1 and H3, influenza B, coronaviruses 229E, HKU1, NL63, OC43, SARS-CoV-2, SARS-CoV-2, parainfluenza viruses 1-4, respiratory syncytial virus A/B (RSV), human metapneumovirus A/B, adenovirus, and rhinovirus/enterovirus; and bacteria: *Mycoplasma pneumoniae, Chlamydophila pneumoniae* and *Bordetella pertussis*). 

Patients were identified by analyzing laboratory registers that noted all patients tested via multiplex RT-PCR. All positive and negative patients were recorded in our database. Eligible cases were determined after analyzing electronic patient records and applying inclusion and exclusion criteria. For each patient, we collected demographic data (age, sex), symptoms at admission, blood test results, treatment received, complications and outcome, and duration of hospitalization. All blood tests were performed in the local NIMCH laboratory; their interpretation was made in relation to the age and sex of the patients, according to the normal ranges indicated by the manufacturer. Any deviation from the normal range was noted as decreased or increased, as appropriate.

To facilitate a more comprehensive examination of the data, the patients in the study were classified into four distinct groups based on their RT-PCR results. The first group consisted of children who tested negative for any of the targeted viruses. The second group comprised children who were positive for rhinovirus but did not exhibit any other respiratory virus infections. The third group included children who were positive for other respiratory viruses besides rhinovirus. The fourth and final group consisted of children who were co-infected with rhinovirus and other respiratory viruses. All comparisons were made in relation to the group of children who were positive for rhinovirus only.

Statistical analysis was performed with IBM SPSS Statistics for Windows, version 25 (IBM Corp., Armonk, NY, USA). The level of statistical significance was set at *p* < 0.05. For continuous variables with normal distribution, we presented mean values, standard deviation, and paired t-test results. For the continuous variables with non-Gaussian distribution, we presented the interquartile range (IQR, defined as 25th percentile and 75th percentile) and the results of the non-parametric Mann–Whitney U test. In analyzing the relationships between categorical dichotomous variables, we used the chi-square test with risk calculation by odds ratio (OR) and 95% confidence interval (95% CI).

## 3. Results

### 3.1. General Data Analysis

**Figure 1:**
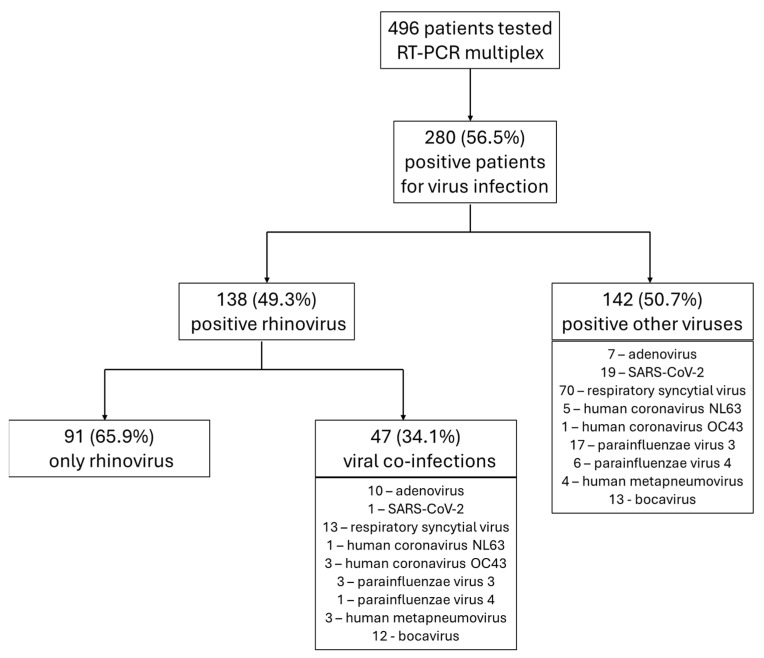
Distribution of cases in the study.

A total of 496 children hospitalized with ILI symptoms underwent RT-PCR testing during the period under review. The positivity rate for at least one viral pathogen was 58.5% (n = 280). Rhinovirus was identified in 138 patients, representing 27.8% of all children tested and 49.3% of all positive samples. In 34.1% (n = 47) of rhinovirus-positive cases, co-infections with other respiratory viruses were identified. The distribution of cases and RT-PCR results is illustrated in Figure 1.

### 3.2. Specific and Comparative Characteristics of Rhinovirus-Positive Cases

**Figure 2:**
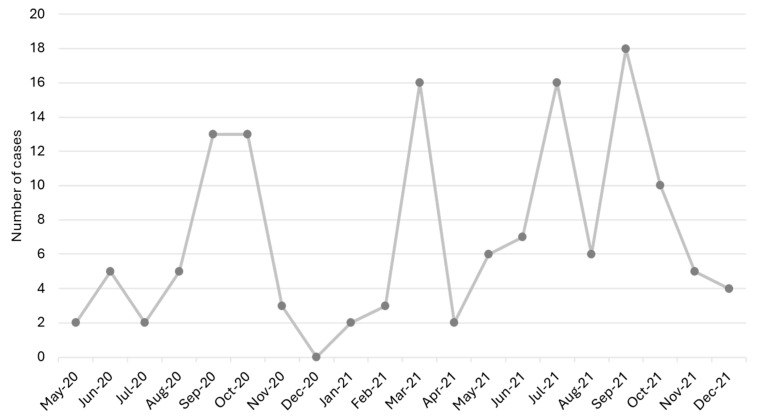
Distribution of rhinovirus cases by month.

Of the 138 children identified with rhinovirus infection, 55.8% (n = 77) were male, and the median age was 12.5 months (IQR: 2, 41.8 months). The temporal distribution of rhinovirus cases, as illustrated in Figure 2, provides a comprehensive overview of the virus’s circulation patterns throughout the year.

**Figure 3:**
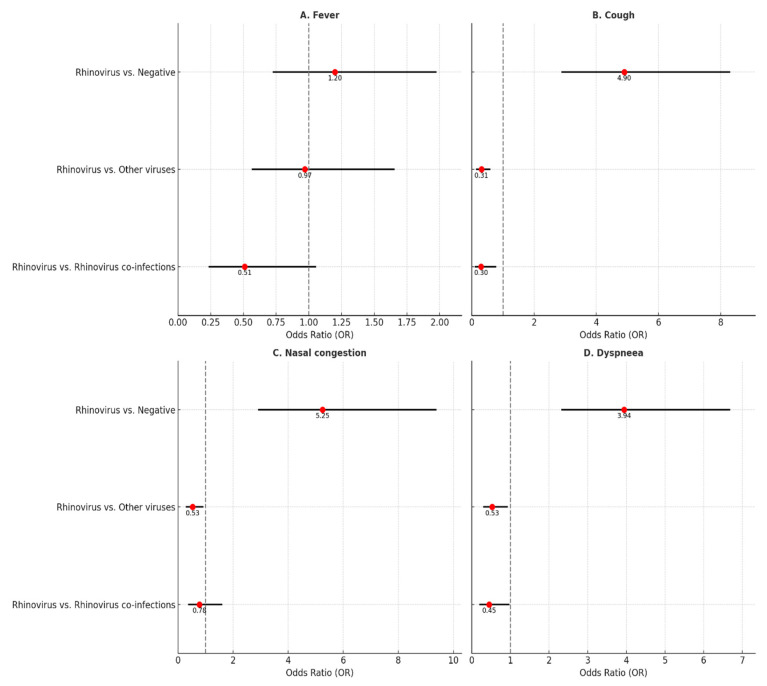
Probability of fever, cough, nasal congestion, and dyspnea in patients with rhinovirus infection compared to other groups.

Cough (63.7%) and dyspnea (51.6%) were the most prevalent symptoms among children hospitalized for rhinovirus infection. However, significant differences were observed in the clinical presentation between the analyzed groups (Table 1). In comparison to negative cases, children with rhinovirus were 4.90 times more likely to present with cough (OR = 4.90, 95% CI: 2.90–8.27), 5.25 times more likely to present with nasal congestion (OR = 5.25, 95% CI: 2.94–9.36), and 3.96 times more likely to present with dyspnea (OR = 3.94, 95% CI: 2.33–6.67), as illustrated in Figure 3 and Table 1 In comparison to children who were positive for other respiratory viruses, the likelihood of cough (OR = 0.31, 95% CI: 0.16–0.57), nasal congestion (OR = 0.53, 95% CI: 0.31–0.90), and dyspnea (OR = 4.90, 95% CI: 2.90–8.27) was significantly lower in those with a rhinovirus infection (Figure 3). The presence of another respiratory virus in conjunction with rhinovirus was associated with an increased frequency of cough (63.7% vs. 85.1%, *p* = 0.009, OR = 0.30, 95% CI: 0.12–0.76) and dyspnea (51.6% vs. 70.2%, *p* = 0.036, OR = 0.45, 95% CI: 0.21–0.96). The prevalence of fever was 47.3% among children with rhinovirus, which did not differ significantly from the other three groups (*p* > 0.05 for each, Table 1, Figure 3).

Analysis of blood test results revealed a 3.52-fold greater likelihood of having increased WBC (OR = 3.52, 95%CI: 1.92–6.45) and a 3.25-fold greater likelihood of having increased neutrophils (OR = 3.25, 95%CI: 1.74–6.06) in children with rhinovirus compared to those who tested positive for other respiratory viruses. Additionally, the risk of elevated CRP was 2.02 times higher (OR = 2.02, 95%CI: 1.18–3.44) in those with rhinovirus than those with other viruses. 

The odds of receiving an antibiotic during hospitalization were found to be 1.92 times lower (OR = 0.52, 95% CI: 0.31–0.86, *p* = 0.011) in children who tested positive for rhinovirus compared to those with negative RT-PCR results. A high proportion of children with rhinovirus (63.7%) received inhalation therapy, and the rate of use increased in children who tested positive for other respiratory viruses (80.3%, *p* = 0.005) and in rhinovirus co-infections (70.2%, *p* = 0.446). Corticosteroids were administered to 42.9% of children with rhinovirus, a frequency that was higher than in those who tested negative (23.1%) or positive for other respiratory viruses (35.9%), but lower than in those with co-infections (55.3%).

**Figure 4:**
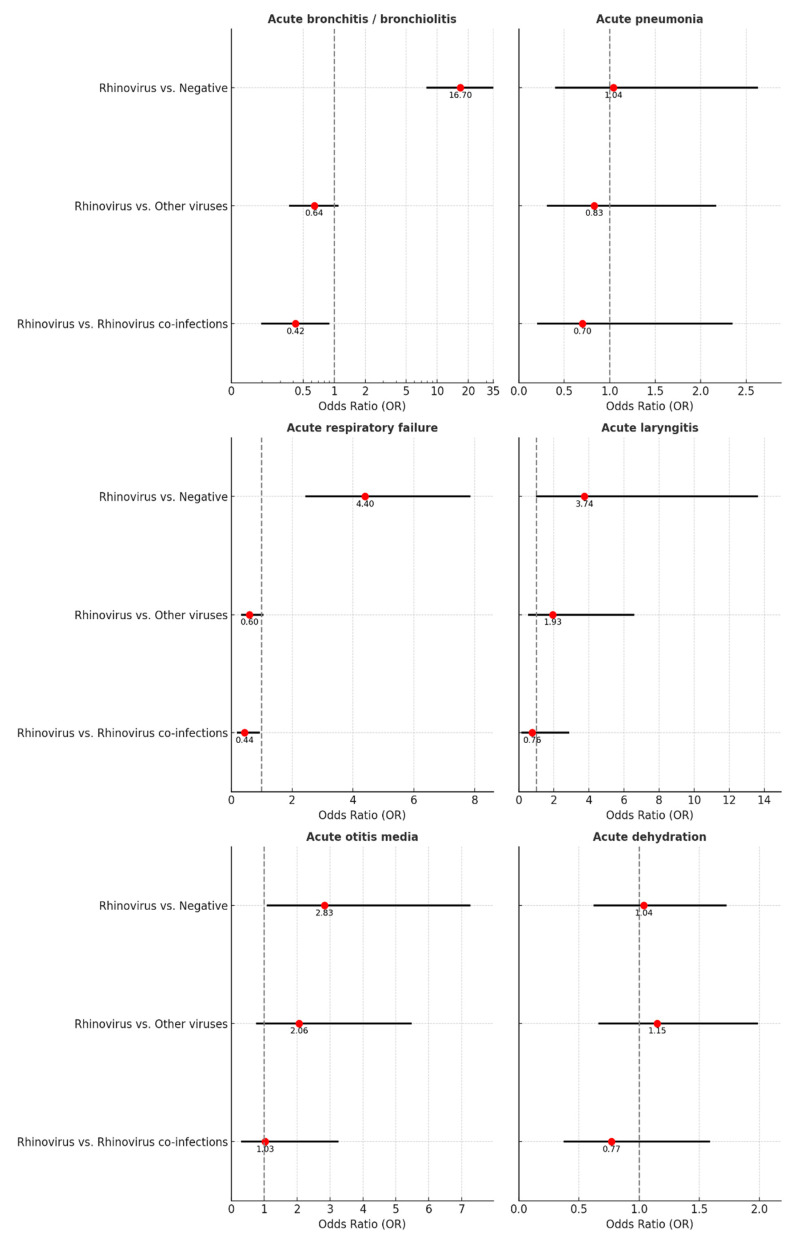
Probability of developing complications in patients with rhinovirus infection compared to other groups.

The risk of acute bronchitis/bronchiolitis was 16.7 times higher in children with rhinovirus compared to children who tested negative (OR = 16.70, 95% CI: 8.02–34.75). In the case of co-infection, the risk decreased by 0.42 times for children infected with rhinovirus (OR = 0.42, 95% CI: 0.20–0.88, Table 1, Figure 4). Similarly, the risk of respiratory failure was 4.40 times higher in rhinovirus-positive children compared to those who tested negative (OR = 4.40, 95% CI: 2.47–7.83), but decreased compared to cases positive for other respiratory viruses (OR = 0.60, 95% CI: 0.35–1.08) or to cases with co-infections (OR = 0.44, 95% CI: 0.21–0.91). Additionally, the risks of acute otitis media (OR = 2.83, 95% CI: 1.11–7.24) and acute laryngitis (OR = 3.74, 95% CI: 1.03–13.59) were significantly elevated in comparison to those without identified viruses upon RT-PCR testing. No differences in the risk of acute pneumonia or acute dehydration were identified (Figure 4, Table 1).

## 4. Discussion

In this retrospective study conducted in one of the largest pediatric hospitals in the capital of Romania, we sought to highlight the clinical characteristics and outcomes associated with rhinovirus infection in children, comparing them with those of children who tested negative or positive for other respiratory viruses. During our analysis, we identified instances of positive infection with the rhinovirus, which occurred concurrently with the advent of the SARS-CoV-2 pandemic. This finding is particularly significant in the context of the broader changes in the epidemiology of respiratory viruses during this period. The SARS-CoV-2 pandemic led to an unprecedented shift in the circulation dynamics of respiratory pathogens, driven largely by public health interventions such as lockdowns, social distancing, and widespread use of masks. Notably, epidemiological surveillance studies have documented a dramatic decline in the incidence of traditionally prevalent respiratory viruses, most strikingly the influenza virus, which saw its incidence drop to near-zero levels in many regions. This shift highlights the complex interplay between rhinovirus and other respiratory viruses during the pandemic and underscores the need for continued monitoring to understand the long-term impacts on viral circulation and infection patterns in pediatric populations [14,15]. Similarly, the incidence of RSV, human coronaviruses, human metapneumovirus, and influenza viruses decreased. As for rhinovirus, a notable decrease in cases only occurred between March and May 2020, with the weekly percentage of positive results for rhinovirus being approximately 14.9% at the end of March and only 3.2% at the end of May. Thereafter, circulation levels increased to pre-pandemic values, with the percentage of positive results in October 2020 being similar to results in other years [16,17,18]. The study conducted by Smedberg et al. in the first part of 2021 demonstrated that 90% of patients presenting with respiratory symptoms specific to a viral infection and testing negative for SARS-CoV-2 but positive for at least one other respiratory pathogen were positive for rhinovirus [19]. In our study, rhinovirus was identified in 49.3% of the pediatric subjects with a positive multiplex RT-PCR result. The closure of educational institutions and the limitation of access to children’s recreational areas also appeared to contribute to the reduction in rhinovirus infections at the onset of the pandemic. However, in the long term, this did not prove to be a significant factor, as an increase in incidence was observed even before the resumption of academic activities [20]. The epidemiological and natural course of respiratory viral infections may be altered by interactions between viruses in cases of co-infection. This occurs through the alteration of receptor expression or other host cell factors that are necessary for replication. Studies have demonstrated that SARS-CoV-2 exhibits a markedly slower growth rate than rhinovirus. In the event of co-infection between the two viruses, the latter will be suppressed. Nevertheless, if rhinovirus infection occurs subsequently, the viral load of SARS-CoV-2 in bronchial epithelial cells is diminished, thereby attenuating the severity of symptoms associated with SARS-CoV-2 [21,22]. 

In our study, the clinical features of rhinovirus infection were characterized by respiratory manifestations that are typical of a viral upper respiratory tract infection. Cough was identified as the most prevalent symptom associated with rhinovirus infection. In comparison to patients infected with other respiratory viruses, children with rhinovirus infection exhibited a significantly reduced likelihood of developing additional respiratory symptoms, including rhinorrhea, wheezing, dyspnea, and fever. These observations are in accordance with the existing literature, which emphasizes that rhinovirus infection is not associated with distinctive pathognomonic signs or symptoms. Consequently, the diagnosis of this infection remains a significant clinical challenge, necessitating the integration of clinical, epidemiological, and laboratory criteria to ensure an accurate diagnosis [6,23,24]. Moreover, instances of asymptomatic rhinovirus infection have been documented, with a prevalence of up to 32% in children under four years of age, and this prevalence decreases with increasing age. However, the concept of asymptomatic rhinovirus infection requires careful interpretation, as advances in molecular diagnostic methods, such as RT-PCR, have revealed the presence of viral genetic material for prolonged periods after remission of the acute clinical episode. Therefore, the identification of viral RNA in an asymptomatic clinical context does not inherently signify an active infection. Rather, it may suggest the persistence of residual viral fragments [23,25]. This finding underlines the importance of correlating laboratory results with the patient`s clinical features to avoid overdiagnosis and mismanagement of pediatric patients.

The analysis of blood tests yielded no significant associations between identified items and the diagnosis of rhinovirus. The frequency of inflammatory syndrome was found to be higher among children with rhinovirus infections than among those with infections from other respiratory viruses. Nevertheless, other studies have demonstrated that rhinovirus monoinfections may be associated with mild to moderate elevations in serum CRP values [26], and that very high increases in CRP may indicate the possibility of a bacterial co-infection or superinfection.

The findings of our study indicate that a positive result on the RT-PCR test, particularly for rhinovirus, was associated with a notable reduction in the rate of antibiotic administration. It is well established that the majority of acute respiratory infections in children are of viral etiology [27,28]. In this context, the excessive and inappropriate use of antibiotics in viral infections, in the absence of bacterial co-infection, contributes significantly to the global phenomenon of antimicrobial resistance, which is a major public health problem [29,30].

The rapid differentiation between viral and bacterial infections is of crucial importance in clinical practice, as it ensures the appropriate administration of treatment. Multiplex RT-PCR assays have been demonstrated to be a valuable tool for this purpose [31]. Multiplex RT-PCR assays allow simultaneous identification of a broad spectrum of respiratory pathogens with high sensitivity and specificity, thereby reducing the need for empiric antibiotic administration in cases of unknown etiology [31]. For instance, a positive result for rhinovirus unambiguously indicates the presence of a viral pathogen and suggests that antibiotics are not indicated unless there is a high clinical suspicion of bacterial superinfection. Numerous studies have shown that, in the absence of molecular diagnostic tools, viral infections are often inappropriately managed with antibiotics, primarily due to the clinical challenges in distinguishing viral from bacterial infections based solely on symptoms and standard laboratory investigations. This diagnostic uncertainty leads to the overprescription of antibiotics, despite the lack of evidence for bacterial involvement, highlighting the critical need for more accurate diagnostic methods to guide appropriate treatment decisions [29,32,33]. The implementation of multiplex RT-PCR technologies in pediatric and emergency medicine units has led to a significant decrease in antibiotic prescriptions, while reducing hospitalization length and healthcare costs [31,34]. In particular, the identification of rhinovirus, one of the most common viral agents causing respiratory infections in children, has been associated with a reduction in antibiotic use of over 30% in cases of upper and lower respiratory tract infections [35], similar to our study, where we identified a reduction in antibiotic consumption compared to children with negative RT-PCR results. This molecular diagnostic approach offers significant advantages at both the individual and population levels. At the individual level, it helps to avoid adverse effects associated with unwarranted antibiotic treatment. At the population level, it contributes to maintaining the efficacy of antibiotics and preventing the spread of antimicrobial resistance.

Finally, our findings revealed that children with rhinovirus infection exhibited a heightened susceptibility to developing acute otitis media, acute respiratory failure, and acute bronchiolitis or bronchitis compared to those who tested negative. Nevertheless, the aforementioned risks are significantly diminished when compared to children who have tested positive for other respiratory viruses. It has been demonstrated that rhinovirus is one of the most prevalent viral pathogens associated with respiratory complications in children, exerting a significant influence on respiratory morbidity in this vulnerable population [36,37]. Although the risk of complications is higher in the presence of rhinovirus than in its absence, this risk is still lower when compared with other respiratory viral infections, such as those caused by RSV or influenza viruses [18,38,39]. In particular, rhinovirus infections are associated with an increased risk of developing acute otitis media, especially in children under the age of 3 years, which may lead to the need for therapeutic intervention and, in some cases, further complications if not treated appropriately [40,41]. Furthermore, clinical data indicate that pediatric patients with rhinovirus-positive respiratory viral infections are more likely to require hospitalization due to respiratory failure, though this likelihood is lower than in cases of RSV infections [42]. Consequently, in this context, the early identification of viral pathogens via advanced molecular assays, such as multiplex RT-PCR, is of paramount importance in order to ascertain the risk of complications and to customize therapeutic management in order to reduce morbidity. The implementation of these tests helps to prevent the overuse of antibiotics and reduce the length of hospitalization, which has a positive impact on the prognosis of pediatric patients.

It is important to acknowledge the inherent limitations of our study in order to contextualize the results. Firstly, the retrospective nature of the analysis may introduce a potential bias in the interpretation of the data, particularly in the assessment of the clinical picture and the evolution of the patients, as the available data may have been subject to variability in the way it was documented. Furthermore, it was not feasible to entirely negate the impact of potential discrepancies in the methodology employed for RT-PCR testing, in addition to the variations in therapeutic strategies applied to the children included in the study. These factors could potentially influence the comparability of the results. Notwithstanding these limitations, our study remains pertinent in terms of the large number of cases analyzed and the rigor of the evaluations performed, thereby contributing to a more nuanced understanding of the clinical and developmental differences between the groups analyzed according to the RT-PCR results.

## 5. Conclusions

We have shown in this study that rhinovirus is one of the most important viral agents involved in acute respiratory tract pathology in children. Clinical and blood test data are not suggestive for positive diagnosis of rhinovirus, and the use of multiplex RT-PCR molecular tests is helpful in clearly establishing the etiology. The implementation of these advanced diagnostic methods can bring significant benefits in practice, as a positive rhinovirus result helps reduce unnecessary antibiotic administration and optimizes patient management, thereby decreasing the risk of severe complications such as acute respiratory failure and acute otitis media.

## Figures and Tables

**Table 1:** Characteristics of children with rhinovirus and comparison with other groups.

Characteristics	Only Rhinovirus Infection	Negative Patients	*p*-Value	Other Respiratory Viruses	*p*-Value	Rhinovirus Co-Infection	*p*-Value
	N = 91	N = 216		N = 142		N = 47	
Demographic data							
Male	49 (53.8)	114 (52.8)	0.862	78 (54.9)	0.862	28 (59.6)	0.521
Age (months), median (IQR)	12 (2, 53)	6.5 (0, 58.5)	<0.001	3 (0, 24.5)	<0.001	13 (4, 32)	0.457
Clinical features							
Malaise	38 (41.8)	73 (33.8)	0.184	56 (39.4)	0.729	19 (40.4)	0.887
Fever	43 (47.3)	92 (42.6)	0.454	68 (47.9)	0.920	30 (63.8)	0.064
Cough	58 (63.7)	57 (26.4)	<0.001	121 (85.2)	<0.001	40 (85.1)	0.009
Nasal congestion	39 (42.9)	27 (12.5)	<0.001	83 (58.5)	0.020	23 (48.9)	0.497
Dyspnea	47 (51.6)	46 (21.3)	<0.001	95 (66.9)	0.019	33 (70.2)	0.036
Diarrhea	18 (19.8)	57 (26.4)	0.219	18 (12.7)	0.143	6 (12.8)	0.303
Vomiting	17 (18.7)	34 (15.7)	0.105	15 (10.6)	0.079	2 (4.3)	0.019
Laboratory findings							
Increased WBC	38 (41.8)	66 (30.6)	0.058	24 (16.9)	<0.001	14 (29.8)	0.169
Decreased WBC	3 (3.3)	16 (7.4)	0.172	10 (7.0)	0.223	0 (0.0)	NA
Increased neutrophils	34 (37.4)	64 (29.6)	0.184	22 (15.5)	<0.001	13 (27.7)	0.254
Decreased neutrophils	1 (1.1)	14 (6.5)	0.076	6 (4.2)	0.251	1 (2.1)	NA
Increased monocyte	55 (60.4)	125 (57.9)	0.680	78 (54.9)	0.406	31 (66.0)	0.527
Increased lymphocyte	9 (9.9)	24 (11.1)	0.751	17 (12.0)	0.624	4 (8.5)	0.828
Decreased lymphocyte	22 (24.2)	36 (16.7)	0.124	34 (23.9)	0.920	9 (19.1)	0.502
Elevated CRP	51 (56.0)	96 (44.4)	0.063	55 (38.7)	0.009	20 (42.6)	0.132
Treatment							
Antibiotics	34 (37.4)	115 (53.2)	0.011	71 (50.0)	0.058	21 (44.7)	0.350
Aerosol therapy	58 (63.7)	52 (24.9)	<0.001	114 (80.3)	0.005	33 (70.2)	0.446
Isotonic saline solution *	9 (9.9)	7 (3.2)	0.023	19 (13.4)	0.423	5 (10.6)	0.892
Hypertonic saline solution *	15 (16.5)	25 (11.6)	0.243	12 (8.5)	0.061	2 (4.3)	0.041
Salbutamol *	35 (38.5)	25 (11.6)	<0.001	30 (21.1)	0.003	14 (29.8)	0.312
Adrenaline *	24 (26.4)	20 (9.3)	<0.001	79 (55.6)	<0.001	21 (44.7)	0.029
Cortisone ^#^	39 (42.9)	50 (23.1)	<0.001	51 (35.9)	0.287	26 (55.3)	0.164
Complications							
AOM	10 (11.0)	9 (4.2)	0.023	8 (5.6)	0.135	5 (10.6)	0.920
Acute bronchitis or bronchiolitis	43 (47.3)	11 (5.1)	<0.001	83 (58.5)	0.094	32 (68.1)	0.019
ARF	36 (39.6)	28 (13.0)	<0.001	74 (52.1)	0.060	28 (59.6)	0.025
Acute laryngitis	6 (6.6)	4 (1.9)	0.042	5 (3.5)	0.346	4 (8.5)	0.734
Acute pneumonia	7 (7.7)	16 (7.4)	0.920	13 (9.2)	0.718	5 (10.6)	0.751
Acute dehydration	35 (38.5)	81 (37.5)	0.862	50 (35.2)	0.617	21 (44.7)	0.479

*—these treatments were administered as aerosol therapy (wet nebulization); ^#^—oral or intravenous administration; IQR—interquartile range; ARF—acute respiratory failure; WBC—white blood cells; CRP—C-reactive protein; AOM—acute otitis media; NA—not applicable.

## Data Availability

The data are available through reasonable request to the corresponding author.
